# Alveolar Abscess

**Published:** 1877-02

**Authors:** G. D. Pollock

**Affiliations:** President of the Pathological Society of London.


### ARTICLE III.

### *Alveolar Abscess, Dependent on Diseased Teeth.*

Read before the Odontological Society of Great Britain, by G. D. Pollock, F. R. C. S., President of the Pathological Society of London.

Mr. President and Gentlemen.—The consideration of Alveolar Abscess can be nothing new to the members of the Odontological Society, but it must always be one of interest, as it is by no means a common occurrence, and is not unfrequently important in its consequences. Moreover, it is a condition which may especially be said to bring together in consultation the dentist and the surgeon; and, as far as my experience goes, the cause of the mischief is too frequently overlooked by the medical practitioner. I hope, therefore, I need offer no apology in making it the subject of my present communication.

I have so frequently had occasion to call the attention of our pupils at St. George's Hospital to this subject, that I have occasionally almost felt that I might be supposed to be riding a hobby too hard. But your President will bear me witness that, with all we have done and said in past years, the recurrence of such cases—I might almost say monthly—proves that the subject is not too generally or fully appreciated; that the cause is frequently overlooked by the medical attendant, and that the patient is consequently permitted to linger on in discomfort, and often with disfigurement.

Alveolar abscess may be considered as of two varieties—one of a superficial and comparatively innocent character, popularly known as gum-boil; the other of a deeper formation, and complicated with various conditions, and what we may truly designate as perforating alveolar abscess. Gum-boil may occur on any side of the alveolus, forms quickly, and usually, when opened, heals as quickly. I do not propose to deal more with this class of cases than to express the opinion that if the abscess recurs frequently, as it is occasionally apt to do, care should be taken to examine thoroughly into the condition of the tooth, and, as regards treatment, to give vent to matter as easily as possible. How far simple inflammation of a healthy tooth, or a defective or carious condition, gives rise to such an abscess, we need not stop to inquire. The more formidable and deep-seated abscess, originating in the alveolar cavity—most often at the extremity of a fang—and which makes its way through the alveolar process, and as a rule outwards—true alveolar abscess, which constantly demands the aid of the surgeon—is the chief subject I wish to draw attention to.

The cause and the mode of origin of alveolar abscess have been well and fully considered by those who have already written on diseases of the teeth. I think, however, it is desirable to refer shortly to some of these observations. Hunter in reference to this says: —"Sometimes deeper abscesses occur than those commonly called gum-*biles*. They are often of very serious consequences, producing carious bone. They commonly arise from disease in the tooth, and more especially in the cuspidati ; these teeth passing further into the jaw than the others. Their depth in the jaw being beyond the attachment of the lip to the gum, if an abscess forms at their points, it more readily makes its way through the common integuments of the face than between the gum and lips, which disfigures the face ; and when in the lower jaw, looks like the evil. In the upper jaw it makes a disagreeable scar on the face, about half an inch from the nose. These although they may sometimes arise from diseases of the teeth and gums, yet are properly the object of common surgery, and the surgeon must apply to the dentist if his assistance is necessary to pull out the tooth, or to perform any other operation which comes under his province."

Fox says :—" Carious teeth frequently become inflamed at the root, and supuration takes place in the socket, attended with swelling and soreness of the gums. In these cases the same laws are observed for the exit of matter as in abscess in general ; viz., ulceration takes place in some part of its surface, so as to make an outlet for the matter in the best possible situation. When matter forms at the root of a tooth, the beriosteum which covers its fang thickens, and in some cases becomes detached from it ; the matter is accumulated as in a bag, by the extension of which considerable pressure is made against the sides of the socket, the consequence of which is, that the part of the alveolar process situated on the outside becomes absorbed, rather than that within the mouth.

Mr. Tomes says :—" With the formation of pus a process is established for effecting its escape. Either the periosteum becomes detached through the whole length of the fang, and the matter is discharged at the neck of the tooth, or, what is much more common, a hole is formed in the wall of the alveolus, through which the pus gets into the gum. In some few cases, in which the inflammation implicates to a considerable extent the adjoining tissues, the abscess, instead of opening in the gum, extends into the cheek and opens on the surface of the face, and through the opening pus continues to be discharged till the tooth is removed."

Mr. Bell remarks that, "of all the diseases which attack the gums and alveolar processes, none is so common, and, perhaps, few are so frequently misunderstood, as that which is commonly termed 'gum-*bile*.' The very name which is popularly given to it is at once a proof of the mistaken notion commonly entertained respecting its nature, and a means of perpetuating the error. The gum is, in fact, only secondarily affected, the cause being invariably seated within the alveolus. I propose, therefore, to call it alveolar abscess, as more correctly designating its true nature and situation. It is produced by various causes. Now and then, though very rarely, it is the result of inflammation of the periosteum of the sound tooth, from cold or some other local cause; it may arise from mechanical injury to a tooth as its being loosened, or partially dislocated by a blow. The irritation of toothache is also a common cause of its occurrence; but by far the most general is the existence of a dead tooth or root, acting as an extraneous body in the socket."

Mr. James Slater says:—"The presence of dentinal caries is its most frequent cause by far, and it may or may not be preceded or accompanied by toothache."

Whether any other causes than caries of a tooth be productive of alveolar abscess I cannot say; but as far as an experience now extending over some years enables me to express an opinion, I can say truly of the many instances which have come to my notice, I have never seen one in which a defective or a carious tooth was not the cause of the mischief. The results of these abscesses are so various, their treatment is so often misunderstood by medical men, their complications and consequences are often so serious, and so constantly call for the interference of the surgeon, that I propose to relate my experience of some of these results.

From all I have observed, it seems to be no means an uncommon circumstance that a patient should suffer no inconvenience from the presence of a decayed tooth, the cause of an alveolar abscess, until some external swelling or disfigure ment, or disagreeable discharge from the nose takes place, and not till then does the patient have recourse to medical advice. It is this absence of toothache, I think, which leads so frequently to the true cause of alveolar abscess, with its consequences, being overlooked. That the formation of the abscess is often almost painless and quite free from toothache I have seen exemplified in several cases. It has often happened to me to observe that when abscess has made its way externally, pain in the affected tooth is the last thing the patient would acknowledge; nor until the appearance of the swelling has the patient been aware of anything wrong. I conclude that, in such cases, the progress of the abscess has generally been very slow; or, perhaps, some partial outlet to the matter has occurred through the decayed tooth, or that the perforation of the outer plate of the alveolus has occurred so early as to have allowed freedom to the pus, and that the patient probably forgot the pain he at first suffered, from its having been but slight.

Matter having once passed through the outer plate, may make its way in various directions, depending somewhat on the position of the decayed tooth and its various surroundings.

I believe the rule holds good that all serious or important results are due to the abscess opening outwards, or upwards when in the upper jaw, the most common course; when it opens inwards it is, as far as I have observed, superficial, and readily heals.

If the abscess has been allowed to open externally, or has been opened by the medical attendant, when pointing in any part externally, and the offending tooth be not removed the abscess soon contracts, but a sinus remains. The orifice of the sinus contracts, but becomes prominent, while the surrounding tissues become thickened and hardened. So that we observe in such cases a small elevated mass of infiltrated tissue, in the centre of which is often a nipple-like point of granulation, through which a fine probe may be passed deep down towards the surface and base of the alveolar process. If the thickened patch be taken between the fingers and attempted to be moved over the jaw, it will be found more or less adherent to the latter, often tied by a kind of string-like process to that part of the jaw in which the diseased tooth lies.

A lady was recommended to consult me by my friend, Dr. Abercrombie, of Cheltenham. She had for two years suffered from an ulcer on the right side of her face, midway and a little posterior to a line drawn from the angle of the mouth to the base of the jaw. For its relief various local measures and much constitutional treatment had been ineffectually resorted to. Suspecting the cause of the ulcer, I examined the mouth, and found a decayed stump in the lower jaw corresponding to the situation of the ulcer. As soon as I alluded to the state of the tooth as its cause, the patient at once expressed her doubts on the point, as she stated she had never suffered trom the toothache. I, however, strongly recommended the removal of the tooth, as it offered the only chance for the ulcer to heal. I heard nothing more of my patient for some weeks, when she called to thank me for my advice. She applied to Mr. Rodgers after she first saw me, and as he entirely confirmed my view of the case, the tooth was removed by him. The ulcer soon healed, but there remained a scar, which time alone could soften, but would never entirely remove.

In another case, after much or little pain about a tooth, a patient will complain of offensive discharge from the nose; and it is usually some assistance to the surgeon to note that this discharge is most distinctly referred to one nostril by the patient.

A gentleman consulted me a few years ago who had been for some two or three years a sufferer from constant offensive discharge from his right nostril. His health was good, his habits temperate, his residence the country ; nor was there any suspicion of syphilis or scrofula. There was a constant disagreeable discharge from the right nostril. When lying down he was conscious of its trickling down the pharynx. He had been under much local and general treatment, with no apparent benefit. After careful examination of the nostril, which gave no evidence of disease, I examined his mouth. I could not detect anything like a carious tooth, but one—the first molar—looked a little more discolored than the rest, I tapped it sharply with the steel handle of a small instrument. This immediately made the patient wince. The tooth was unnaturally tender, and by my advice it was removed. A few days after he called to report improvement. The tooth produced was dark-colored, with its fangs inflamed. At the end of six weeks he had lost all sense of the discharge from the nostril, and when I saw him some time subsequently, he had quite recovered from this very disagreeable condition.

I could record other cases of abscess in the antrum, the result of decayed teeth, but this one case is sufficient to illustrate my statement, that much discomfort may occur with a defective tooth without the patient being cognizant of toothache.

The cases related may be considered comparatively simple in their results; but the effects of alveolar abscess are not always so. The following cases will prove my statement: A man was admitted into St. George's Hospital, under my care, with a large brawny swelling on the right side of his neck, extending from the surface of the jaw to the clavicle. It had encroached so much on the median line in front that the larynx was much pushed to the left side, and this produced a good deal of difficulty in breathing. He could not open his mouth to allow a proper examination of the teeth; but I suspected a bad tooth to be the cause of the mischief. Two incisions were made into the inflamed tissues with some relief. The following day there was more difficulty in breathing, and it was necessay to open the trachea to save life. The operation was rendered somewhat difficult by the displacement of the larynx. The swelling now began to subside, as there was free discharge from the wounds; and as soon as the mouth could be sufficiently opened, Mr. Vasey removed a decayed molar from the lower jaw, evidently the cause of all this mischief. The patient left the hospital quite well. But a short time ago I had to see a lady late one evening, with a large hard swelling between the chin and thyroid cartilage. She had some difficulty in breathing, and considerable difficulty in swallowing. A free incision in the median line allowed the escape of some very offensive pus, with great relief to her symptoms. I now examined her teeth ; one lower incisior was painful on being tapped, and she consented to have it removed as soon as the swelling caused by the abscess had subsided. On removal, the extremity of the fang was found to be carious. After this she remained well for more than a year, when she again sent for me with another abscess in the same situation. After the abscess was opened, the left middle incisior, which was tender on being tapped, was also removed ; and she remained well when last seen, some time subsequently.

Hunter, with his careful observation, has remarked that when abscess occurs in the lower jaw, it often "looks like the evil" ; a condition to which, I conclude, we should apply the term "scrofulous" in these days. A well-marked and interesting case illustrative of Hunter's observation came under my notice at St. George's Hospital some few years ago. A comparatively healthy young woman was sent to me for a terribly bad ulcerated neck. From the base of the jaw, on the right side, to near the clavicle, was a mass of indurated, dark-colored, and thickened skin and cellular tissue, with numerous ulcerated openings on the surface. To a less extent the left side of the neck was similarly affected. I was asked to recommend her for a bed at the Margate Infirmary, as it was supposed she was suffering from scrofulons of the neck. I may state that the parts were so indurated, ulcerated, and so dark red in color that the general appearance was much more characteristic of the brawny condition of cancerous infiltration with ulceration than anything of scrofulous nature. I was not, however, satisfied as to the character of this mischief, and made an examination of her mouth. On both sides of the lower jaw were numerous decayed stumps. I suggested their removal, and she was handed over to the dresser of the weak to be relieved of these stumps. To my great satisfaction, in the space of a short time, with very simple local applications, all the ulcers healed, and the integuments of the neck assumed a natural healthy condition. About a year or eighteen months after this, she again applied to me for some recurrence of ulceration on the left side of the neck. I looked in the mouth and saw two stumps remaining in the left lower jaw. I taxed her with not having carried out the instructions I formerly gave, viz., to have all the stumps removed ; upon which she replied, that she had had so many removed on that occasion, and as she had suffered a great deal of pain, she thought it would do no harm to allow these two to remain. She at once had them removed, and her recovery was as rapid as it was satisfactory. Mr. James Slater relates a somewhat similar case, and quotes another recorded by M. Robert, in which suppuration extended to the shoulder and upper part of the breast, followed by death ; and in which "it was shown that the suppuration had originated at the angle of the jaw, immediately in contact with the decayed wisdom tooth."

There are conditions, however, which, stopping short of the formation of matter, may prove formidable enough, which require the surgeon's careful attention and the early removal of the offending tooth.

Two cases of a most interesting character will illustrate my statement: Some few years ago I was asked to see a patient suffering severely from supposed disease of the right eye. He was suffering excessive pain. The right eyeball was so much protruded that the lids could not close over it, and in consequence of the exposure cornea was becoming hazy and opaque. The eyeball itself was not very vascular, the pupil was widely dilated, and sense of vision entirely gone. I could not detect my disease in the eye itself to account for this protrusion. I carefully examined the orbit, but could not satisfy myself of the presence of any growth, nor could I feel anything like fluctuation from cyst or abscess. It occurred to me that some extraneous mischief might possibly account for the protrusion, as there was nothing in the condition of the globe or the orbit to explain it, the dilatation of pupil and loss of sight being probably due to the mechanical pressure on the eyeball and the stretching of the optic nerve. I made a careful examination of the bones surrounding the orbit, with a view to ascertain if any ossifice implication might explain these conditions. On passing my finger down the side of the nose, the patient winced when it reached a point midway between the inner canthus and angle of the mouth. Immediately suspected a tooth might account for this, and on examination of the mouth found a stump in the upper jaw on the right side, and a molar next it which had been stopped. He made no complaint of toothache. It appeared to me almost too hopeful to expect that these defective teeth could possibly be the cause of such serious mischief; but as an operation had been suggested for the condition of the eye, it was agreed that these teeth should be first removed before any other operative measures where had recourse to. The following day Mr. Vasey removed both stumps and stopped tooth. The fangs were inflamed and thickened, but there did not appear to be sufficient to account for such an amount of mischief.

The result was most satisfactory; pain immediately commenced to subside and the protrusion to diminish. At the end of a week the patient was able to close the lids over the cornea, and the latter entirely recovered its natural lustre. At the end of a fortnight it was difficult to say that there had been anything the matter with the eye, except that the pupil was still somewhat dilated, and insensible to the stimulus of light. Subsequently, though vision was not restored, the pupil acted properly in account with that of the sound eye.

Some two years subsequently to this attack the patient called on me, and stated that he had pain at the back of the left orbit—the opposite side, and as he was about to be absent from England for some time, he wished my advice on this point. He now mentioned to me that some time prior to the attack in the right orbid, but that, as they were not very severe or persistent, he paid no further attention to them. On looking into his mouth a decayed molar was seen in the upper jaw on the left side. This was removed with perfect relief to the orbital pain, and the patient has had no further trouble in this respect. Mr. James Salter has published a very interesting case of amaurosis, consequent on acute abscess of the antrum, produced by a decayed tooth, in a communication to the Medico-Chirurgical Society, in which he did me the honor to refer to the case just related.

Another case, but less serious in its consequences, occurred to me three years ago. A lady residing in the South of France became subject to acute neuralgic pains about the right orbit and side of the head. This was gradually followed by some general thickening of the soft tissues below and around the the inner margin of the right orbit, and generally spreading outwards. There was also decided prominence of the globe when I saw her, a few months after commencement of these symptoms, some slight double vision at times, and much headache. I could observe nothing in the condition of the eye, or the contents of the orbit, to account for the slight prominence of the former, beyond the general vascularity and the chronic thickening of soft tissues, with slight periostial enlargement of the inner halves of the margins of the orbit. On examining the teeth, a molar in the right upper jaw was seen to be defective and discolored, and in front was a stopped bicuspid. By my advice the molar was removed Mr. Formansell, who then recollected that he had drilled the body of this tooth some time previously; the aperture was still patent, and the tooth appeared defective and discolored.

The result, though partially beneficial, was not entirely satisfactory; the patient still complained of some pain, and suggested the removal of the stopping from the other tooth, as she thought it rather sensitive. This was done, and now steady, gradual improvement took place, and the patient has remained well since, with perfect recovery of vision.

A dentigerous cyst will occasionally be the seat of suppuratiou. A patient was admittted into the St. George's Hospital under my care a few months ago, with an enlargement of the lower jaw on the right side, which on examination, appeared to involve the substance of the bone. All the teeth (the three molars) over it had been removed previously to admission. On examination of the mouth a small orifice, from which pus exuded, was observed in front, between the cheek and the gum, and in the floor of the sulcus; a probe prssed readily into a large cavity, but no bone or exposed tooth could be felt. The sac was freely laid open inside the cheek; the finger was passed into the cavity, which was felt to be lined by a smooth membrane, but no exposed bone or foreign substance could be felt. The cavity was dressed in with lint dipped in a strong solution of sulphate of copper. It rapidly diminished in size, and was gradually filled up when the patient returned home.

The cases related are of sufficient importance, I trust, to justify the time taken up by their description; and, I think, indicate fairly the very great necessity of attention being early given to the state of the mouth in all cases of ulcers, sinuses, or swellings in the neighborhood of the jaws. I would almost go the lengh to say that very few are the ulcers in this situation which have an independent origin; but that they are, in the great majority of cases, due to defective teeth and subsequent alveolar suppuration.

As to treatment I have but a few words to say, and it agrees entirely with the advice given by Hunter, Fox, Bell, Tomes and Salter. The rnle is as simple as it is certain in its effects; the tooth or stump must be at once removed. In many cases I have seen the removal of the tooth sufficient to evacuate the matter locked up, and would recommend this to be done always in the first instaece if possibly. When, however, this operation does not effect the purpose, and the abscess has not made for itself any other outlet, it should be at once freely incised within the mouth.

The removal of the tooth will not, however, always prevent an abscess, once started, from becoming troublesome subsequently. In a case in which I was lately consulted, the offending tooth had already been removed—a molar from the right side of the lower jaw. But much induration remained about the outer surface of the bone. The patient was a young lady in rather delicate health; and thought a free incision was made between the cheek and the jaw, matter continued to collect, and ultimately presented externally, and was obliged to be let out by means of a small external puncture. This soon closed, and the patient experienced no further annoyance. When a sinus has long been permitted to exist without the extraction of the tooth, it is very apt to continue to discharge long after the removal of the offending tooth. It becomes lined by a semi cuticular membrane, and if fluid be injected, it will run into the mouth. Mr. Bell has recorded an interesting case of this kind, and I have quite lately seen one in which the patient used cotton-wool to plug the orifice. In such cases it is best at once to freshen the edges of the puckered opening and bring them together by fine silver sutures, an operation usually followed by direct and permanent closure of the sinus.

I have met with a few cases in young persons in which a cyst has formed at the bottom of a defective tooth; and which, on being laid open, has disclosed the apex of the fang projecting into the cavity. On the removal of the tooth this point has been found carious. The cyst has always contracted and filled up after the removal of the tooth.

I looked upon these cysts as differing in character from those known as dentigerous cysts, and as dependent on a necrosed condition of the fang. Ticdouloureux, or neuralgia, is, I believe, frequently to be attributed to the presence of decayed teeth, and such instances go to prove how indefinite in position and intensity are the pains dependent on such causes.

In conclusion, I would only add that the cases which eave been related teach us this lesson—that in all instances of abscess or ulcer in the uppe.ı or lower maxillary regions, in certain cases of discharge from the nostrils, or in pain about the orbits, with defective teeth in the jaws, it is aiways best at once to have recourse to their removal; acting on the simple principle that it is best to clear away any doubtful poiut in the treatment of a case before recourse is had to any other measurers; for the maxim of "an empty house is better than a bad tenant" applies equally to surgery as to commence.—*Dental Miscellany.*

